# Spatial and chemical heterogeneity of insect cuticular hydrocarbon layers: insights from extraction methods and acclimation regimes

**DOI:** 10.1098/rsos.261519

**Published:** 2026-11-04

**Authors:** Sascha Schlüter, Kiara Mangin, Florian Menzel

**Affiliations:** Institute of Organismic and Molecular Evolution, Johannes-Gutenberg-Universität Mainz, Mainz, RP, Germany

**Keywords:** cuticular hydrocarbons, insects, phase behaviour, molecular interactions, lipid layers, melting behaviour, formicidae, *Schistocerca*, *Acheta*, *Periplaneta*

## Abstract

Insect cuticles are covered by a complex mixture of cuticular hydrocarbons (CHCs) that are essential for desiccation resistance and chemical communication, yet the physical structure of CHC layers remains poorly understood. We extracted CHCs from five insect species (desert locusts, house crickets, American cockroaches and two ant species) using solvents of differing polarity and extraction durations, and from insects reared under different temperature regimes. Extractions with *n*-hexane were enriched in relatively solid CHCs (*n*-alkanes and terminally branched monomethylalkanes), while extractions with dichloromethane and dichloromethane–methanol yielded proportionally more fluid compounds (internally branched monomethyl and dimethylalkanes, unsaturated hydrocarbons). Solid-phase CHCs were proportionally more abundant in shorter extractions, whereas fluid-phase compounds were proportionally more abundant in prolonged or sequential extractions. These patterns are consistent with a model of cohesive solid domains surrounded by more accessible fluid regions, though this interpretation remains indirect and alternative explanations (e.g. differential solubility and diffusion kinetics) cannot be excluded. CHCs of similar phase behaviour covaried across treatments, indicating that they aggregate within shared domains. Warm acclimation increased the proportion of solid compounds, whereas fluctuating temperatures enhanced CHCs with intermediate phase behaviour. Our results suggest spatial and chemical heterogeneity in the CHC layer, with patterns of differential extractability consistent with clustering into phase-distinct domains, though direct imaging is required to confirm spatial topology. This potential heterogeneity may facilitate CHC multi-functionality, with the phase behaviour and domain membership of each CHC depending on its biophysical properties relative to the individual's overall CHC profile.

## 1. Introduction

The evolution of multi-functional traits poses a central challenge in evolutionary biology, as they are simultaneously subject to multiple, often conflicting, selection pressures from ecological, physiological and sexual contexts [1,2]. Cuticular hydrocarbons (CHCs), which cover the cuticle of probably all insects, exemplify such traits. They form a primary barrier against desiccation [3–5] and mediate chemical communication [6–8]. Further, less studied functions include protection against pathogens, footpad adhesion, self-healing of cuticle abrasions and joint lubrication [9–12].

CHC profiles are chemically diverse, typically comprising up to 100 compounds per individual. These include straight-chain *n*-alkanes, mono- to tetra-methylated alkanes, alkenes, alkadienes and, in rare cases, methylated alkenes, with carbon chain lengths generally spanning C20–C50 [13,14].

The physical properties of individual CHCs are determined by their molecular structure: chain length, degree of branching and unsaturation. Straight-chain *n*-alkanes pack tightly into ordered arrangements with relatively high melting points (40–60°C for C18–C43 chains) and can thus be described as ‘more solid’ under biologically relevant temperatures [6,15,16]. Branching and double bonds disrupt molecular packing, lowering melting points by up to 50°C relative to equivalent *n*-alkanes, making these compounds ‘more fluid’ at ecologically relevant temperatures [16,17]. Throughout, we use ‘more solid’ and ‘more fluid’ in a relative sense: a compound is ‘more solid’ if it has a higher melting point (or, if liquid, a higher viscosity) than the others in the same profile, and ‘more fluid’ if the reverse. We adopt this relative terminology deliberately, as precise melting points are unknown for most insect CHCs, because viscosity is temperature-dependent and undefined for solids, and because a compound that is solid in isolation may be liquid if in a mixture (e.g. owing to freezing-point depression). Generally, *n*-alkanes and terminally branched alkanes are ‘more solid’, whereas alkenes, alkadienes and multiply branched alkanes are ‘more fluid’, with internally branched monomethylalkanes (intMonoMe) in between. For CHC mixtures, as a whole, these differences translate into broad melting ranges extending from below –45°C to between +30 and +45°C for the last solid components, consistent with biphasic or multi-phasic behaviour at ecologically relevant temperatures (15–35°C) [6,16].

These physical properties link directly to biological function. More solid, tightly packed CHC layers minimize cuticular water loss by reducing permeability [3–5,9,18], while more fluid components facilitate the diffusion and perception of semiochemicals for chemical communication [6–8]. Fluid CHCs also contribute to the self-healing of cuticle abrasions [9], the adhesion of footpads [10] and joint lubrication [11]. The coexistence of solid and fluid phases within a single CHC layer, a biphasic or multi-phasic organization, thus appears essential for CHC multi-functionality, enabling insects to simultaneously maintain a waterproofing barrier and support chemical signalling [6,7,19,20]. CHC composition is not fixed. Within an individual, profiles adjust plastically through acclimation over days to weeks, whereas between species, evolutionary divergence shapes more permanent differences. Both tend in the same direction: warmer or drier conditions are generally associated with more solid, saturated profiles and cooler or wetter conditions with more fluid ones, although between-species variation also reflects habitat and ecological pressures beyond temperature and humidity alone [1,21–24]. Mutualistic interactions can further shape CHC profiles, as observed in parabiotic ant species [23].

Support for biphasic organization comes from multiple lines of evidence. Gibbs’ phase-separation model predicts that CHCs segregate into coexisting solid and liquid phases, with solid (crystalline) domains providing structural stability and liquid regions enabling semiochemical mobility [6,7]. Synchrotron attenuated total reflectance Fourier-transform infrared (ATR-FTIR) microspectroscopy has revealed coexisting solid and liquid phases at the microscale on *Periplaneta americana* and *Gryllus bimaculatus* cuticles [25]. Complementary *in situ* X-ray scattering measurements on *P. americana* further confirm that most lipids remain liquid even at lower temperatures [26]. Microrheological measurements on two ant species demonstrated coexisting gel and liquid phases within the CHC layer, with the fluid fraction showing bimodal viscosities (comparable to honey: 600–1000 mPa s, and with olive oil: 100–150 mPa s) and with warmer-acclimatized individuals developing more viscous layers [27]. Monte Carlo simulations of thin hydrocarbon films support phase separation, with longer chains adsorbing to the underlying solid surface and forming ordered, solid-like layers, while shorter chains accumulate at the outer (film–vapour) surface and remain fluid [28]. Consistent with this, intermediate-chain *n*-alkanes spanning the CHC size range are known to undergo surface freezing, the formation of an ordered, crystalline monolayer at the film interface at temperatures above the bulk freezing point, providing an interfacial route to solid-domain stabilization that does not depend on a bulk melting-point shift [29]. Despite this evidence, a critical question remains unresolved: how are solid and fluid phases spatially organized across the cuticle surface? Whether CHCs form laterally distinct domains, vertical layers or some combination of both (figure 1) is not yet established—yet this arrangement determines whether an insect can simultaneously waterproof its cuticle and present mobile signalling compounds, making the spatial organization central to how CHCs reconcile their conflicting functions.

**Figure 1.**
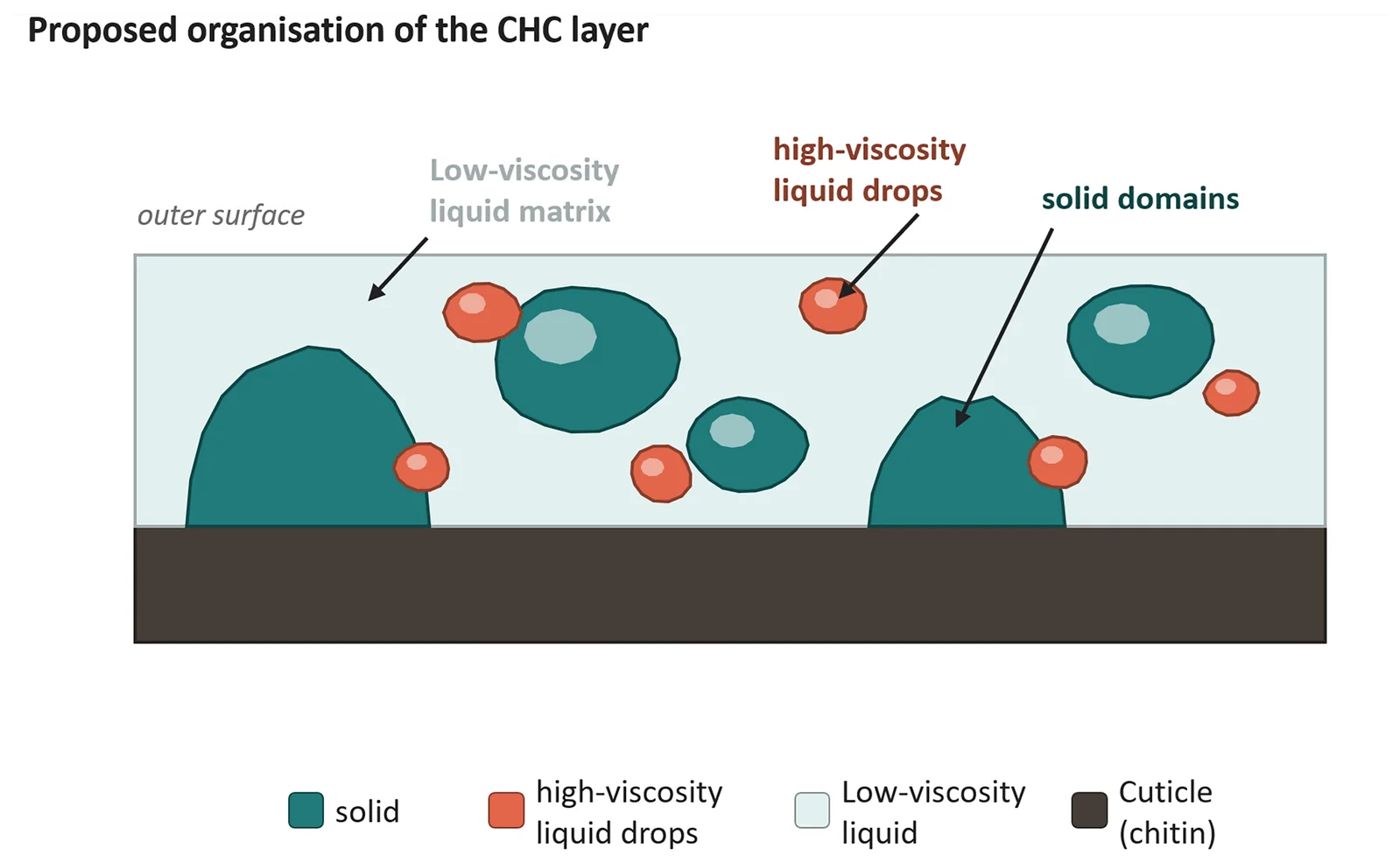
Hypothetical biphasic organization of the CHC layer. Schematic representation of the CHC layer illustrating a proposed biphasic model in which solid CHCs (teal) form discrete island- or iceberg-like domains embedded within a surrounding matrix of low-viscosity, liquid CHCs (pale) and high-viscosity drops (orange). Together, these phases constitute the continuous CHC layer that overlays the cuticle (dark-brown). This model suggests spatial heterogeneity in CHC physical state, which may influence barrier properties and surface dynamics.

To address this question, we used differential extraction as an indirect approach to probe spatial and chemical heterogeneity in the CHC layer, working with five insect species spanning a wide range of CHC profiles: *Schistocerca gregaria*, *Acheta domesticus*, *P. americana*, *Lasius fuliginosus* and *Formica rufibarbis*. We varied extraction solvent (*n*-hexane, dichloromethane and a dichloromethane–methanol mixture) and extraction duration (10 and 20 minutes and a sequential ‘10 + 10’ protocol), and applied thermal acclimation treatments (20 and 30°C, fluctuating) to three of the five species. Based on the ‘like dissolves like’ principle [30] and anticipated differences in CHC mobility or matrix embedding, we expected solvents of differing polarity to show differential affinity for CHC classes of different phase behaviour, and extraction duration to reflect differences in CHC accessibility within the layer. Critically, our goal was not to identify an optimal extraction method, but to use method-dependent differences to gain insights into the physical and spatial organization of the CHC layer. Our specific aims were: (i) to determine how extraction conditions (solvent type and duration) alter recovered CHC profiles, thereby revealing information about spatial and chemical heterogeneity within the CHC layer, (ii) to examine how thermal acclimation modifies CHC composition, and (iii) to identify which CHC classes covary across experimental manipulations, as covariation indicates shared biophysical properties or spatial proximity within the layer. We employed a multi-species approach to assess consistency of patterns across diverse CHC profiles, while acknowledging that treatments were not applied uniformly across all species (acclimation in three non-social species; extraction duration in two ant species). Rather than studying all potential treatments in only a few model organisms, we preferred to investigate multiple species to see how far our results hold across different insects. We anticipated that solvent- and duration-dependent extraction differences, acclimation-driven composition shifts and CHC covariation patterns would provide convergent evidence for whether CHC layers exhibit heterogeneous, phase-separated spatial organization rather than a uniform mixture.

## 2. Material and methods

### 2.1. Study species and rearing

We selected five insect species for our study based on their divergent compositions of CHC profiles, which are representative of a broad range of insect species and can be readily obtained in larger quantities (figure 2). The species used were *S. gregaria* (Orthoptera: Acrididae; desert locust, henceforth abbreviated as ‘*Sgre*’), *A. domesticus* (Orthoptera: Gryllidae; house cricket, henceforth ‘*Adom*’), *P. americana* (Blattodea: Blattidae; American cockroach, henceforth ‘*Pame*’), *L. fuliginosus* (Hymenoptera: Formicidae; Jet-black ant, ‘*Lful*’) and *F. rufibarbis* (Hymenoptera: Formicidae; Red-bearded ant, ‘*Fruf*’). We used wingless, subadult desert locusts and adults for all other species. The desert locusts, house crickets and cockroaches were acquired in 2023 from commercial suppliers (*Adom*, *Sgre*: Fressnapf, Mainz, Germany; *Pame*: Schaben & Co., Hückeswagen, Germany) and housed in plastic boxes (21 × 29 × 15 cm) with a 10 cm diameter grid for ventilation on the lid, with each box containing 30 individuals. For *Adom* and *Pame*, box floors were lined with absorbent tissue paper and equipped with egg carton shelters, while *Sgre* boxes contained a layer of fine sand with similar shelters. Both group were fed ad libitum, with crickets and cockroaches receiving carrots and salmon-based cat food, while locusts were provided with freshly plucked grass semi-weekly. Workers of both ant species (*Lful* and *Fruf*) were collected from the wild, each from a single colony to ensure individuals had experienced similar environmental conditions. *Fruf* was collected on the Johannes Gutenberg University Mainz campus (September 2024); *Lful* was collected in 2022 from a colony in the Ober–Olmer Forest near Mainz, Germany. Ant workers were not reared in the laboratory but were immediately frozen at –20°C after collection.

**Figure 2.**
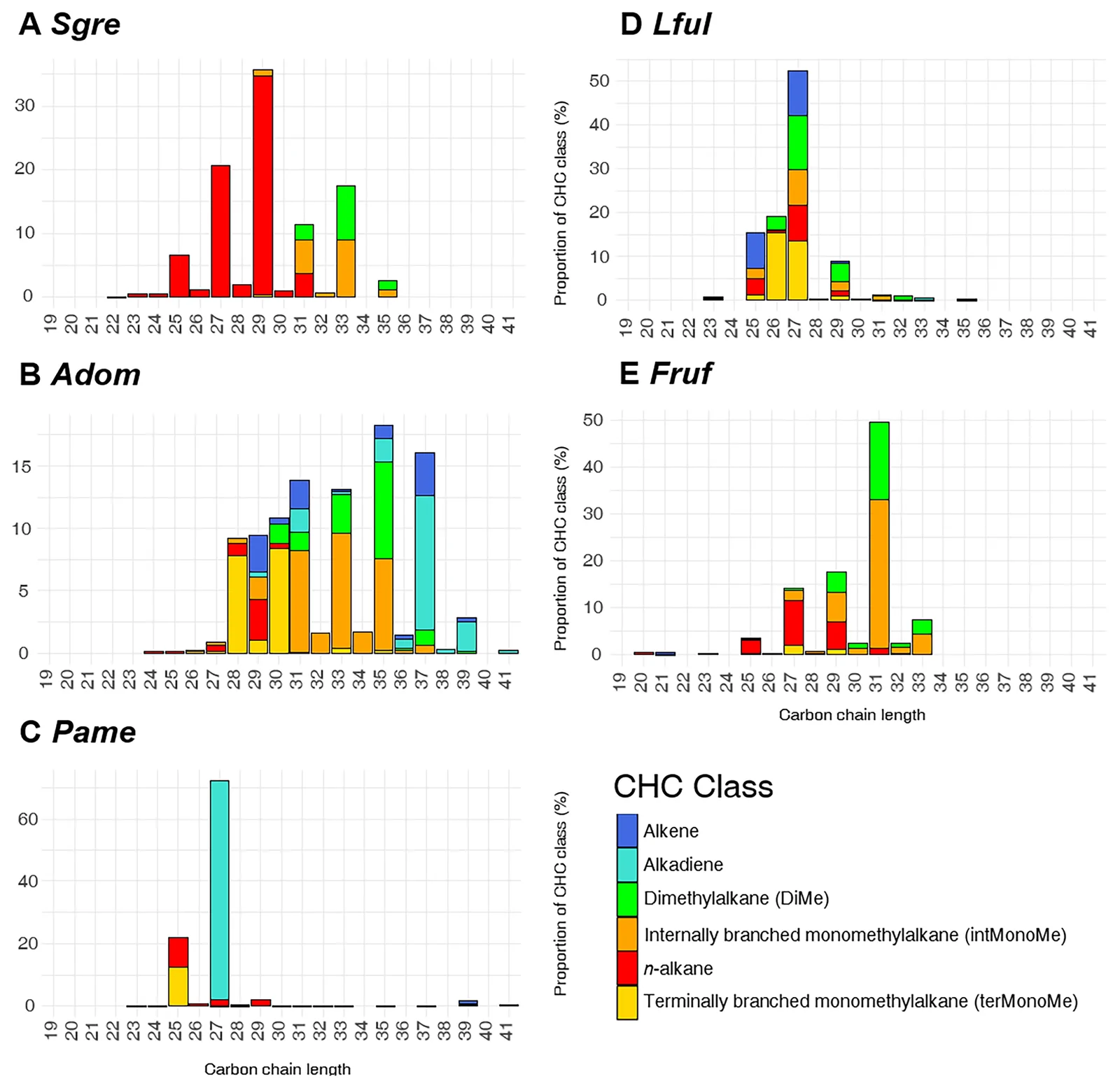
CHC composition in the five study species. (A) *Schistocerca gregaria* (*Sgre*), (B) *A. domesticus* (*Adom*), (C) *P. americana* (*Pame*), (D) *L. fuliginosus* (*Lful*) and (E) *F. rufibarbis* (*Fruf*). The stacked bar plots illustrate the relative abundance (in per cent) of different CHC classes (*n*-alkanes, alkenes, alkadienes, intMonoMe, terminally branched monomethylalkanes (terMonoMe) and dimethylalkanes (DiMe) plotted against carbon chain length (C19–C41). Each bar represents the total proportion of a CHC class for a given chain length, with colours indicating distinct CHC classes. The data represent CHC profiles averaged across all available samples per species (i.e. pooled across all treatments tested for that species).

### 2.2. Experimental design and treatments

We analysed CHC profiles across five insect species, applying up to three treatment factors: solvent type, extraction duration and acclimation temperature. Not all treatments were applied to all species (see table 1 for an overview). Each species × treatment combination was replicated 10 times (*n* = 10).

**Table 1.** Overview of treatments per species. All extractions used immersion in specified solvents/times; acclimation durations 10–14 days. Hex: *n*-hexane, DCM: dichloromethane, DCMM: DCM and methanol.

species	abbreviation	acclimation comparisons (20 and 30°C, fluctuating)	solvent comparisons (Hex, DCM, DCMM)	extraction times comparisons (10, 20 and 10–10 minutes)	replicates per treatment
***S. gregaria***	*Sgre*	Yes	Hex, DCM, DCMM	no	10
***A. domesticus***	*Adom*	Yes	Hex, DCM, DCMM	no	10
***P. americana***	*Pame*	Yes	Hex, DCM, DCMM	no	10
***L. fuliginosus***	*Lful*	No	Hex, DCM, DCMM	yes (plus three solvent types)	10
***F. rufibarbis***	*Fruf*	no	Hex, DCM, DCMM	yes (only with Hex)	10

#### 2.2.1. Acclimation treatments

To assess plastic responses of CHC composition to temperature, *Sgre*, *Adom* and *Pame* were subjected to one of three acclimation regimes in climate-controlled cabinets: constant 20°C, constant 30°C or a fluctuating regime (30°C from 10.00 to 18.00, 20°C from 22.00 to 06.00, with gradual 4-hour ramps between temperature extremes). Acclimation lasted 10–12 days for *Adom* and *Pame* and 14 days for *Sgre*. Insects were killed by freezing at −20°C, with most individuals processed during the morning (approximately 09.00–11.00).

#### 2.2.2. Solvent treatments

To probe the phase-dependent extractability of CHCs, we used three solvents spanning a polarity gradient: *n*-hexane (Hex; non-polar, dielectric constant *ε_r_* ≈ 1.9, linear chain; purity greater than or equal to 95%, Merck KGaA), dichloromethane (DCM; moderately polar, *ε_r_* ≈ 9.1, compact tetrahedral geometry; purity greater than or equal to 99.9%, Carl Roth GmbH + Co. KG) and a 2 : 1 v/v mixture of DCM and methanol (DCMM; methanol *ε_r_* ≈ 33; DCM and methanol purities greater than or equal to 99.9%, Carl Roth GmbH + Co. KG). The DCMM mixture was prepared following Folch [31]. These solvents were selected because their differences in polarity and molecular geometry were expected to result in differential affinity for CHC classes of distinct phase behaviour, following the ‘like dissolves like’ principle [30,32]: non-polar Hex was expected to preferentially associate with more solid, straight-chain or terminally branched compounds, while the more polar solvents were expected to access more fluid, internally branched or unsaturated CHCs. We emphasize that our goal was not to identify an optimal extraction method, but to exploit method-dependent differences to infer the physical and spatial organization of the CHC layer.

#### 2.2.3. Extraction duration treatments (*Lful* and *Fruf*)

To assess whether CHC fractions of differing phase behaviour differ in their extractability over time, we applied three extraction duration treatments to *Lful* and *Fruf*: 10 and 20 minutes and a sequential ‘10–10’ protocol. In the 10–10 protocol, the individual was first extracted for 10 minutes. This first extract was discarded, and the individual was then placed in a fresh vial with the same solvent for a further 10 minutes. The second extract (10–10) therefore contained only compounds that had not been dissolved during the initial 10 minutes, allowing us to target fractions of lower accessibility or solubility.

For *Lful*, a fully balanced design was used (all three solvents × all three extraction durations). For *Fruf*, a reduced design was used: *n*-hexane extractions at all three durations (10 and 20 minutes, 10–10), plus 10-minute extractions with all three solvents. This reduction was justified because preliminary analyses showed non-significant solvent × duration interactions in most cases (table 2), indicating that the main effects of solvent and duration could be assessed adequately without full factorial replication.

**Table 2.** Abundance of different CHC classes in extracts with different extraction times, acclimation treatments and solvents. The table shows *F* and *p*-values from linear models on relative CHC class proportions, fitted separately for each CHC class (*n*-alkanes, terMonoMe, intMonoMe, DiMe, alkenes and dienes) and insect species (*Sgre*, *Adom*, *Pame*, *Lful* and *Fruf*). Models included ‘acclimation’ temperature (20 and 30°C, fluctuating) or ‘extract_time’ (10, 20 and a second 10 minutes extract) as factor 1 and ‘solvent’ (hexane, dichloromethane and dichloromethane-methanol) as factor 2. Data were based on *n* = 10 replicates per treatment combination (individual insects). Proportions were transformed where necessary to achieve normally distributed residuals (‘lm_trans’: none, log(x+1), sqrt or logit). No analyses were performed if a CHC class contained only one or no compounds in the profile. For *Fruf*, separate unifactorial models were used owing to the reduced design. *p*-values below 2.2e-16 are reported as ‘< 2.2e-16’. NA: no results available.

			n-alkane			terMonoMe			intMonoMe			DiMe			alkene			diene		
species	d.f.	effect	lm_trans	*F*	*p*	lm_trans	*F*	*p*	lm_trans	*F*	*p*	lm_trans	*F*	*p*	lm_trans	*F*	*p*	lm_trans	*F*	*p*
*Adom*	2	acclimation	none	**11.54**	**9.03e^–^^5^**	none	**17.48**	**2.41e^–^^6^**	none	**6.24**	**0.004**	none	**19.17**	**9.52e^–^^7^**	log	**12.57**	**4.59e^–^^5^**	none	**3.69**	**0.033**
	2	solvent	none	**6.10**	**0.005**	none	**7.24**	**0.002**	none	0.94	0.396	none	0.47	0.629	log	0.76	0.474	none	1.11	0.339
	4	accl*solvent	none	1.26	0.300	none	1.26	0.301	none	3.60	**0.013**	none	0.97	0.432	log	1.53	0.211	none	**2.71**	**0.042**
*Pame*	2	acclimation	none	**32.96**	**1.53e^–^^9^**	none	**5.11**	**0.010**	sqrt	**4.73**	**0.014**	sqrt	0.07	0.933	none	**5.86**	**0.005**	none	**17.60**	**2.25e^–^^6^**
	2	solvent	none	**20.12**	**5.72e^–^^7^**	none	**27.14**	**1.85e^–^^8^**	sqrt	1.51	0.231	sqrt	1.67	0.200	none	**10.19**	**2.24e^–^^4^**	none	**36.63**	**3.62e^–^^10^**
	4	accl*solvent	none	2.32	0.072	none	2.64	**0.046**	sqrt	1.18	0.332	sqrt	2.34	0.069	none	0.59	0.672	none	3.13	**0.023**
*Sgre*	2	acclimation	none	**24.37**	**5.18e^–^^9^**	none	**74.60**	**< 2.2e^–^^16^**	none	**27.35**	**8.38e^–^^10^**	none	**16.76**	**8.12e^–^^07^**	no alkenes	NA	NA	no dienes	NA	NA
	2	solvent	none	**20.80**	**5.13e^–^^8^**	none	2.42	0.096	none	**19.1**0	**1.60e^–^^07^**	none	**20.86**	**4.92e^–^^8^**	no alkenes	NA	NA	no dienes	NA	NA
	4	accl*solvent	none	0.96	0.431	none	0.96	0.435	none	1.10	0.364	none	1.13	0.348	no alkenes	NA	NA	no dienes	NA	NA
*Lful*	2	extract_time	sqrt	**43.73**	**1.32e^–^^13^**	none	**16.46**	**1.00e^–^^6^**	none	2.51	0.087	none	**32.31**	**4.82e^–^^11^**	none	**27.97**	**5.81e^–^^10^**	one diene	NA	NA
	2	solvent	sqrt	**10.42**	**9.39e^–^^05^**	none	2.75	0.070	none	**6.92**	**0.002**	none	**7.60**	**9.46e^–^^4^**	none	2.11	0.128	one diene	NA	NA
	4	time*solvent	sqrt	1.10	0.363	none	1.59	0.185	none	1.06	0.380	none	**3.11**	**0.020**	none	2.04	0.096	one diene	NA	NA
*Fruf*	2	extract_time	logit	0.98	0.384	logit	3.0737	0.056	none	0.48	0.621	none	**4.82**	**0.012**	none	0.2316	0.794	no dienes	NA	NA
	2	solvent	logit	1.18	0.315	logit	**4.9114**	**0.012**	none	0.16	0.851	none	0.77	0.468	none	2.5689	0.087	no dienes	NA	NA

### 2.3. Cuticular hydrocarbons extraction

Insects were fully immersed in solvent for the specified duration. Vial sizes were matched to body size: 1.5 ml glass vials for *Fruf* and *Lful*, 4 ml vials for *Adom* and 10 ml vials for *Sgre* and *Pame*. After extraction, insects were removed, and the extract was concentrated under a gentle stream of nitrogen gas to approximately 20 µl, then transferred to 1.5 ml vials fitted with micro-inserts.

A total of 2 µl of each extract was injected into a gas chromatograph (7890A, Agilent Technologies, Santa Clara, CA, USA) operating at 250°C in splitless mode, fitted with a Zebron ZB-5HT Inferno capillary column (30 m × 0.25 mm × 0.25 µm; Phenomenex Ltd, Aschaffenburg, Germany). Helium carrier gas was maintained at 1.2 ml min^−1^. The temperature programme started at 60°C, ramped at 60 K min^−1^ to 200°C, then at 4 K min^−1^ to 320°C and was held for 10 minutes. Mass spectrometric detection (5975C, Agilent Technologies) used 70 eV electron ionization, scanning *m/z* 40–550. Data were processed with mass selective detector (MSD) ChemStation software (E.02.02.1431, Agilent Technologies).

Chromatographic peaks were manually integrated. Hydrocarbons were identified using a retention index based on a homologous series of *n*-alkane standards and diagnostic ions following Carlson *et al.* [33]. Compounds were excluded if they were non-hydrocarbons, their maximum relative abundance across all samples of a species was below 0.5% and mean relative abundance below 0.1%, or they occurred in fewer than 20% of samples within any species-treatment combination.

### 2.4. Statistical analyses

All analyses were performed on relative proportions: CHC peak areas were normalized per sample to sum to one, as no internal or external standard was used. Individual compounds were assigned to one of six structural classes: *n*-alkanes, terminally branched monomethylalkanes (terMonoMe; methyl group at position less than or equal to 5), intMonoMe (position 6 and above), dimethylalkanes (DiMe), alkenes and alkadienes. This distinction reflects the known decrease in melting point with increasing methyl group position (positions 2–5) that plateaus at positions 6 and above [16], such that terMonoMe compounds have phase behaviour closer to *n*-alkanes. Within each sample, compound proportions within a class were summed to give a single class-level proportion for use as the response variable.

For each CHC class, a linear model was fitted with the two treatment factors and their interaction as fixed effects (lm(proportion ∼ factor 1 × factor 2), where factor 1 was acclimation temperature for *Sgre*, *Adom* and *Pame* and extraction duration for *Lful* and *Fruf*, factor 2 was solvent type in both cases). For *Fruf*, owing to the reduced experimental design in which extraction duration was only tested with *n*-hexane, separate single-factor models were fitted for each factor independently. Prior to model fitting, response variables were transformed as needed to achieve approximate normality of residuals. Transformations were tested in the following order of preference: none, log(x + 1), square root and logit; the first transformation for which the Shapiro–Wilk test on model residuals yielded *p* > 0.01 was retained. If no transformation achieved this threshold, results were recorded as undetermined and excluded from inference. The significance of both main effects and their interaction was assessed simultaneously using a Type II ANOVA on the full interaction model (Anova, package *car*). Interaction terms were rarely significant (table 2); we therefore performed pairwise post hoc comparisons on the additive model (after removal of the interaction term), using Tukey's contrasts (*glht* with *mcp*, package *multcomp*). Significance was summarized as compact letter displays (levels sharing a letter: adjusted *p* ≥ 0.05).

Covariation among individual CHC compounds within each species was analysed by hierarchical cluster analysis. For each species, we first recorded every compound as its proportion of the total profile within the sample, as described above. We then rescaled each compound across samples so that its values summed to one over all samples of that species; this gave rare and abundant compounds equal weight. Using these rescaled values, we computed the Euclidean distance between every pair of compounds—reflecting how similarly their proportions varied across samples—and grouped compounds by hierarchical clustering, visualized by dendrograms (Ward's minimum-variance method, ward.D2; packages *vegan* and *dendextend*). The number of clusters (*k* = 3 for *Sgre* and *Pame*; *k* = 6 for *Adom, Lful* and *Fruf*) was determined by visual inspection of the full dendrogram. To test whether CHC class or chain length category (short: C19–C29 and long: C30–C41) was non-randomly distributed across clusters, *χ*² tests were applied to the corresponding contingency tables.

To quantify inter-individual variability, we calculated the coefficient of variation (CV = s.d./mean) of each CHC-class proportion within treatment groups (greater than or equal to 3 individuals and class mean greater than 0.5%) and averaged across groups. To assess how much compositional variation was attributable to species identity, we ordinated all individuals by CHC-class composition (non-metric multi-dimensional scaling based on Bray–Curtis distances) and tested for differences among species with PERMANOVA (*adonis2*, 999 permutations; package *vegan*).

## 3. Results

All results described below are based on relative abundances (proportions of total CHC area per sample). An overview of species-level CHC profiles, averaged across all samples per species, is shown in figure 2. Inter-individual variability in CHC-class proportions was low to moderate within treatment groups (median coefficient of variation 31%; supplementary material, table S1), lowest for the dominant class in each species and highest for classes present only in traces. This within-species variation was minor relative to differences among species: across all individuals, species identity alone explained 87% of the variation in CHC-class composition (PERMANOVA on Bray–Curtis distances, pseudo-F₄_,_₃₃₃ = 542, *R*² = 0.87, *p* = 0.001; supplementary material, figure S2).

### 3.1. Solvent effects

Solvent type significantly affected the relative proportions of CHC classes in most species (table 2, *p* < 0.05 for nearly all classes and nearly all species), with the largest differences observed between hexane and the two polar solvents (DCM and DCMM).

Effects were most pronounced in *Sgre* (table 2). *N*-alkanes were most abundant in hexane extracts, intermediate in DCM and lowest in DCMM (Tukey's post hoc *p* < 0.001 for all pairwise comparisons). In *Pame* and *Sgre*, *n*-alkane proportions were significantly lower in DCMM than in DCM. DCM and DCMM did not differ in the remaining species (figure 3).

**Figure 3.**
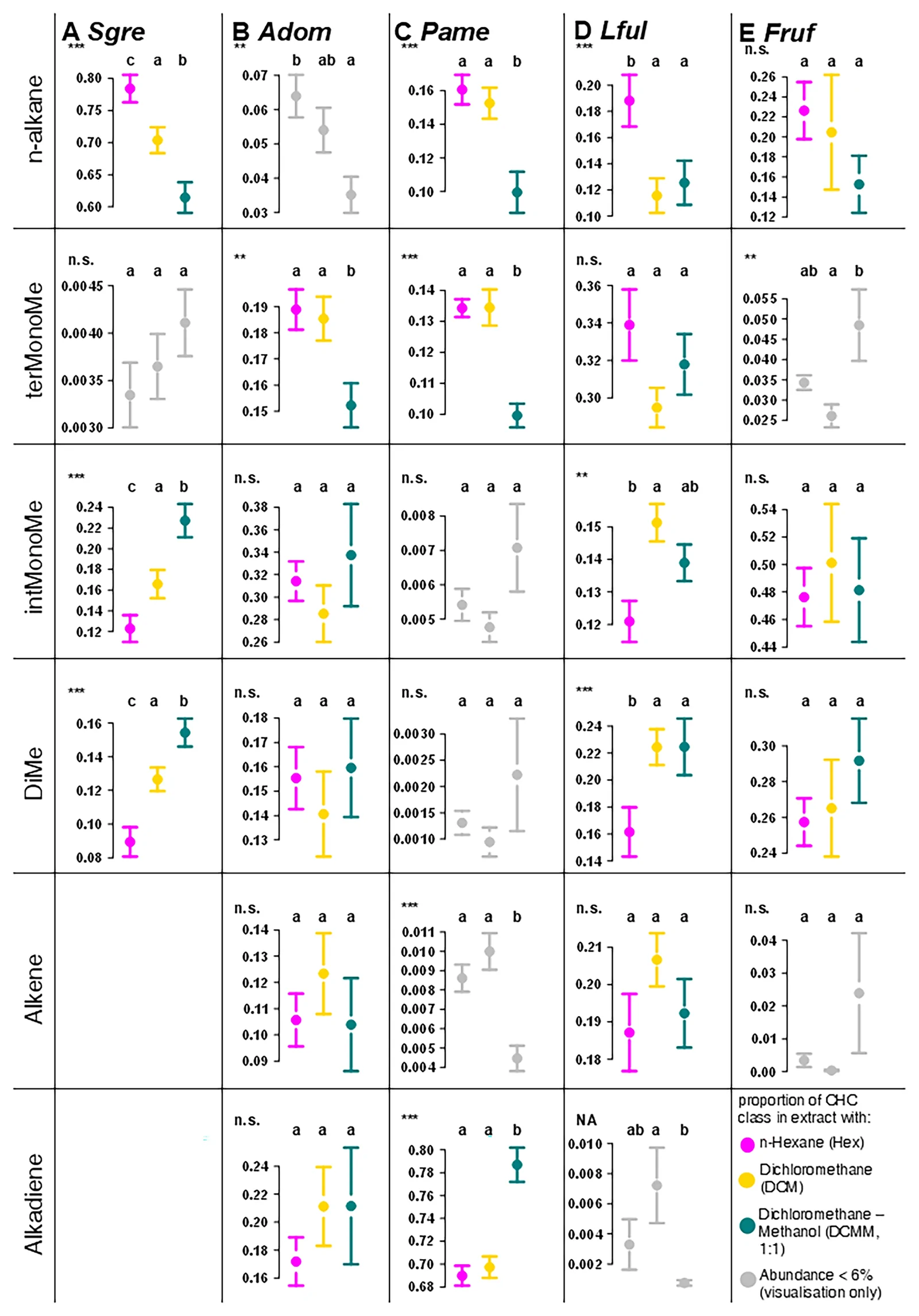
Effect of solvent type on extract composition. The plots show mean ± standard error of the relative abundance of different CHC classes (*n*-alkanes, terMonoMe, intMonoMe, DiMe, alkenes and alkadienes) across three solvent types (Hex, DCM and DCMM) for the five study species: (A) *Sgre*, (B) *Adom*, (C) *Pame*, (D) *Lful* and (E) *Fruf*. Each panel corresponds to a specific CHC class, with plots coloured by solvent type (magenta for Hex, gold for DCM and teal for DCMM). Means are based on *n* = 10 replicates per solvent treatment (individual insects), pooled across available extraction durations for each species (primarily 10 minutes; time-specific effects in ants are shown separately in figure 4). CHC classes with average abundance below 6% are shown in grey for easier visualization but were analysed the same way. Asterisks indicate whether the overall effect of solvent was significant according to ANOVA (***p < 0.001, **p < 0.01, *p < 0.05, n.s. *p* > 0.05). Plots with the same letters are not statistically different according to Tukey post hoc tests (*p* > 0.05).

The terMonoMe showed heterogeneous solvent effects. In *Adom* and *Pame*, terMonoMe followed the same pattern as *n*-alkanes (highest in hexane), while effects were weak and largely non-significant in the other species.

The intMonoMe showed contrasting patterns across species. In *Sgre*, intMonoMe proportions increased progressively from hexane to DCM and were highest in DCMM. In *Lful*, the pattern was reversed, with the lowest intMonoMe proportions in hexane, the highest in DCM and intermediate levels in DCMM.

DiMe proportions were highest in DCMM and lowest in hexane in both *Sgre* and *Lful* (table 2, *p* < 0.001 for both species).

In *Pame*, alkene proportions were highest in DCM, intermediate in hexane and lowest in DCMM (table 2); a similar but non-significant trend was apparent in *Lful*. Solvent effects on alkenes were non-significant in *Adom* and *Fruf*. Alkadienes were absent in *Sgre* and *Fruf* and were present at less than 1% relative abundance in *Lful*. In *Pame*, alkadienes were most abundant in DCMM extracts and significantly lower in hexane and DCM extracts (non-significant in *Adom*).

Alkene and dimethylalkane proportions did not vary in parallel across solvents (i.e. their solvent responses were not correlated), indicating compound class-specific differences in solubility and possibly matrix interactions.

Within species, solvent effects were in the same direction for some CHC classes but opposite for others. In *Pame*, *n*-alkanes, terMonoMe and alkenes all showed similar patterns (highest in hexane), whereas alkadienes showed the opposite pattern. In *Adom*, *n*-alkanes and terMonoMe showed similar patterns; other classes did not show consistent relationships. In *Sgre*, *n*-alkane proportions were highest in hexane, while terMonoMe, intMonoMe and DiMe were highest in polar solvents, showing the reverse pattern. In *Lful*, *n*-alkanes and terMonoMe were most abundant in hexane, while intMonoMe, DiMe, alkenes and alkadienes showed the reverse pattern. Across species, the more solid CHC classes (*n*-alkanes and terMonoMe) were proportionally higher in hexane extracts, whereas the more fluid CHC classes (intMonoMe, DiMe, alkenes and alkadienes) tended to be proportionally higher in DCM or DCMM extracts. The dividing line between these groups differed among species and was most clearly defined in *Sgre*.

### 3.2. Effects of acclimation treatments

Acclimation temperature influenced CHC composition in both compound- and species-specific ways (table 2). Comparisons between constant temperature treatments showed the most consistent pattern in *Sgre*: *n*-alkane proportions were higher in warm-acclimatized (30°C) individuals, while terMonoMe, intMonoMe and DiMe proportions were lower at 30°C than at 20°C (figure 4). *Adom* showed a similar trend for *n*-alkanes, terMonoMe, intMonoMe and DiMe, with the additional observation that alkenes and alkadienes were also at higher proportions in warm-acclimatized individuals. In *Pame*, the pattern was broadly similar, except that alkadienes were at lower proportions under warm conditions (rather than higher, as in *Adom*).

**Figure 4.**
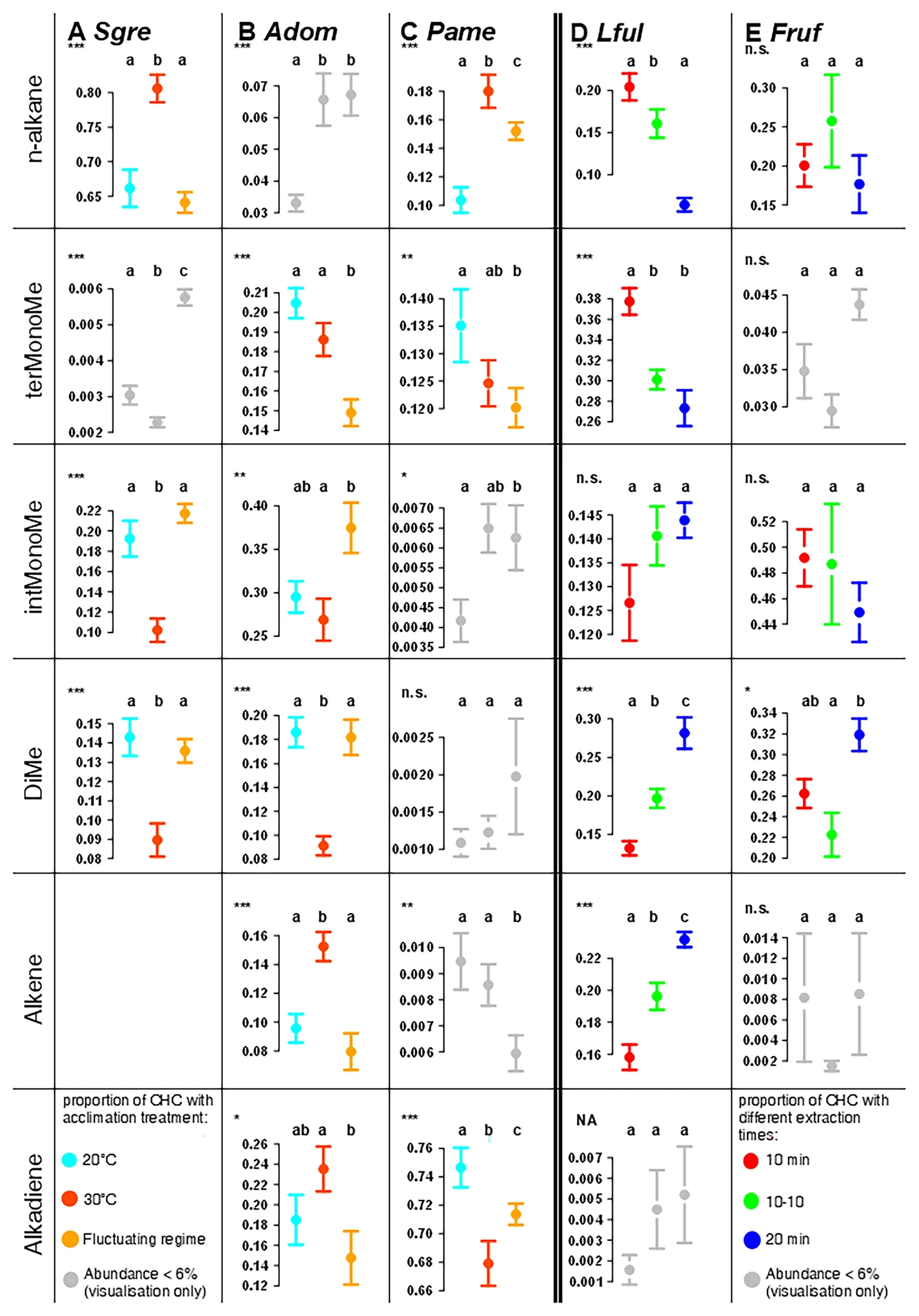
Effect of acclimation temperature and extraction time on extract composition. The plots show mean ± standard error of the relative abundance of different CHC classes (*n*-alkanes, terminal monomethylalkanes (terMonoMe), internal monomethylalkanes (intMonoMe), DiMe, alkenes and alkadienes). Panels A–C (left column) show results for acclimation regimes in *S. gregaria* (A; *Sgre*), *A. domesticus* (B; *Adom*) and *P. americana* (C; *Pame*) across three conditions: 20°C constant (cyan), 30°C constant (orange-red) and fluctuating (orange). Panels D–E (right column) show results for extraction time in *L. fuliginosus* (D; *Lful*) and *F. rufibarbis* (E; *Fruf*) across three protocols: 10 (red), 20 (green) and a second 10 minutes extract (blue; sequential extraction in fresh solvent after discarding the initial 10 minutes extract). Means are based on *n* = 10 replicates per treatment combination (individual insects). All data shown are from extractions with *n*-hexane (standard 10-minute duration for acclimation panels; respective durations for extraction time panels; methods). CHC classes with mean abundance below 6% are shown in grey for easier visualization but were included in all statistical analyses. Asterisks indicate the overall significance of the treatment effect according to ANOVA (***p < 0.001, **p < 0.01, *p < 0.05, n.s. *p* > 0.05). Bars sharing the same letter are not significantly different according to Tukey's post hoc tests (*p* > 0.05).

When fluctuating-temperature profiles were compared with both constant treatments, fluctuating conditions generally produced CHC profiles more similar to those at 20°C than to those at 30°C. In *Sgre*, this was most evident for *n*-alkanes, intMonoMe and DiMe. TerMonoMe, a class with intermediate phase behaviour in *Sgre*, showed higher proportions under fluctuating than under either constant treatment. In *Adom*, fluctuating-temperature profiles resembled those at 20°C for DiMe, alkenes and alkadienes; in *Pame*, fluctuating and 20°C profiles were most similar for alkadienes. For terMonoMe, fluctuating profiles were partially aligned with 20°C conditions, though the pattern was less consistent.

In some cases, fluctuating conditions produced CHC proportions more similar to those at 30°C. In *Adom*, this was the case for *n*-alkanes; in *Pame*, for *n*-alkanes, terMonoMe and intMonoMe. In a smaller number of cases, fluctuating conditions produced the most extreme proportions observed across all treatments: in *Adom*, both monomethylalkane classes reached their highest proportions under fluctuating conditions; in *Pame*, alkenes were at their lowest proportions under fluctuating conditions. In *Sgre*, terMonoMe reached its highest proportions under fluctuating conditions.

### 3.3. Extraction time effects

Extraction duration significantly influenced CHC extract composition, most clearly in *Lful* (table 2; figure 4). More solid CHC classes (*n*-alkanes and terMonoMe) were most abundant after 10 minutes, at intermediate levels after 20 minutes and at the lowest proportions in the 10–10 extracts. By contrast, more fluid CHC classes (intMonoMe, DiMe, alkenes and alkadienes) showed the opposite pattern: lowest proportions after 10 minutes, intermediate after 20 minutes and highest in the 10–10 extracts. This pattern is consistent with differences in extractability between CHC fractions of differing phase behaviour (e.g. owing to compound mobility, matrix binding or cohesion).

In *Fruf*, extraction duration effects were weaker and largely non-significant across CHC classes (table 2). The only significant response was in alkenes, which are present at low proportions in this species. Directional trends in other classes were subtle: *n*-alkane proportions were similar between 10 minutes and 10–10 extracts, terMonoMe proportions were slightly higher in 10–10 extracts, intMonoMe proportions were slightly lower in 10–10 extracts, and DiMe showed a modest increase with sequential extraction. Overall, extraction duration effects were species-specific, with clear and consistent temporal separation in *Lful* and much weaker effects in *Fruf*.

### 3.4. Covariation among cuticular hydrocarbons

Hierarchical cluster analyses revealed that individual CHC compounds showed non-random covariation patterns within each species, grouping by CHC class and, in several species, by chain length (tables 3 and 4). Covariation by CHC class was statistically significant in *Adom*, *Sgre*, *Fruf* and *Lful*, but not in *Pame* (table 4). Covariation by chain length (short: C19–C29 and long: C30–C41) was significant in *Adom*, *Sgre* and *Lful*, but not in *Fruf* or *Pame* (table 4).

**Table 3.** Covariation among CHCs as revealed by cluster analysis. The table shows the number of different CHCs per CHC class assigned to each cluster. Cluster analyses were done for each species separately. The table gives the number of compounds per CHC class (alkenes, alkadienes, DiMe, intMonoMe, terMonoMe, *n*-alkanes and unknown CHCs) and cluster, as well as the number of long (greater than C30–C41) and short (C19–C29) compounds per cluster. See table 4 for the results of *χ*² tests, where we tested whether CHC class composition differs among clusters. CHCs were grouped into six clusters for *Adom, Lful* and *Fruf* and three clusters for *Sgre* and *Pame*.

species	cluster ID	*n*-alkane	terMonoMe	intMonoMe	DiMe	alkene	alkadiene	unknown	long	short
***Sgre***	**1**	4	0	0	0	0	0	0	0	4
	**2**	6	1	1	0	0	0	0	2	6
	**3**	0	0	4	3	0	0	0	7	0
***Adom***	**1**	3	0	0	0	0	1	0	0	4
	**2**	0	6	9	11	1	1	0	21	7
	**3**	4	1	1	0	3	1	0	4	6
	**4**	0	0	0	1	0	0	0	1	0
	**5**	0	1	0	0	0	0	0	1	0
	**6**	0	2	0	0	4	6	0	12	0
***Pame***	**1**	9	3	4	1	1	4	0	9	13
	**2**	0	0	0	1	0	0	0	1	0
	**3**	0	0	1	1	0	1	0	3	0
***Lful***	**1**	0	0	0	0	1	0	0	0	1
	**2**	6	1	0	0	0	0	0	0	7
	**3**	0	7	6	6	3	0	0	2	20
	**4**	0	0	1	0	0	0	0	1	0
	**5**	0	2	0	0	1	1	0	4	0
	**6**	1	0	2	1	0	0	0	4	0
***Fruf***	**1**	1	0	0	0	0	0	0	0	1
	**2**	1	0	0	0	2	0	0	0	3
	**3**	6	0	0	0	0	0	1	2	5
	**4**	0	5	8	20	0	0	0	17	16
	**5**	0	1	4	0	0	0	0	0	5
	**6**	0	0	0	1	0	0	0	1	0

**Table 4.** Non-randomness in distribution of CHC classes and CHC chain lengths across clusters. The table presents results of *χ*² tests (*χ*² statistic, degrees of freedom (d.f.) and *p*-value) assessing whether the distribution of CHC compounds across clusters deviates from random expectation with respect to CHC class (*n*-alkanes, alkenes, alkadienes, intMonoMe, terMonoMe, DiMe, etc.) or chain length category (short: C19–C29 and long: C30–C41). Tests were conducted separately for each species using the compound-to-cluster assignments from hierarchical clustering (Ward's method on double-normalized data; *k* = 3 for *Sgre* and *Pame*, *k* = 6 for *Adom*, *Lful* and *Fruf*). Sample size for each test is the total number of retained CHC compounds (peaks) per species after filtering (methods).

	CHC class			chain length		
	*χ*²	d.f.	*p*	*χ*²	d.f.	*p*
*Sgre*	**16.8**	**6**	**0.0099**	**13.0**	**2**	**0.0015**
*Adom*	**68.8**	**25**	**5.78e^–^^6^**	**19.8**	**5**	**0.0014**
*Pame*	12.3	10	0.26	4.7	2	0.094
*Lful*	**53.1**	**25**	**8.56e^–^^4^**	**30.0**	**5**	**1.46e^–^^5^**
*Fruf*	**91.6**	**25**	**1.57e^–^^9^**	9.7	5	0.084

Across species, *n*-alkanes formed distinct clusters in *Adom*, *Fruf*, *Sgre* and *Lful* (table 3; in *Lful*, shorter and longer *n*-alkanes were grouped together). IntMonoMe and DiMe consistently covaried with each other across all five species, clustering together into a single shared group. TerMonoMe showed variable association, grouping with intMonoMe/DiMe in some species and with *n*-alkanes in others. In *Adom* and *Fruf*, alkenes and alkadienes occasionally covaried with *n*-alkanes. In *Sgre*, which lacks alkenes, *n*-alkanes and DiMe formed separate clusters. In *Pame*, covariation was less structured: only a small number of dominant compounds (including the highly abundant C27-alkadiene) showed clear cluster membership, while minor compounds showed weaker patterns—probably because so few compounds reach high abundance in this species, which increases measurement error and weakens any detectable covariation. Covariation patterns were similar when cluster analyses were conducted separately for each acclimation treatment (figure 5 for *Sgre* and *Adom*; supplementary material, figure S1 for other species).

**Figure 5.**
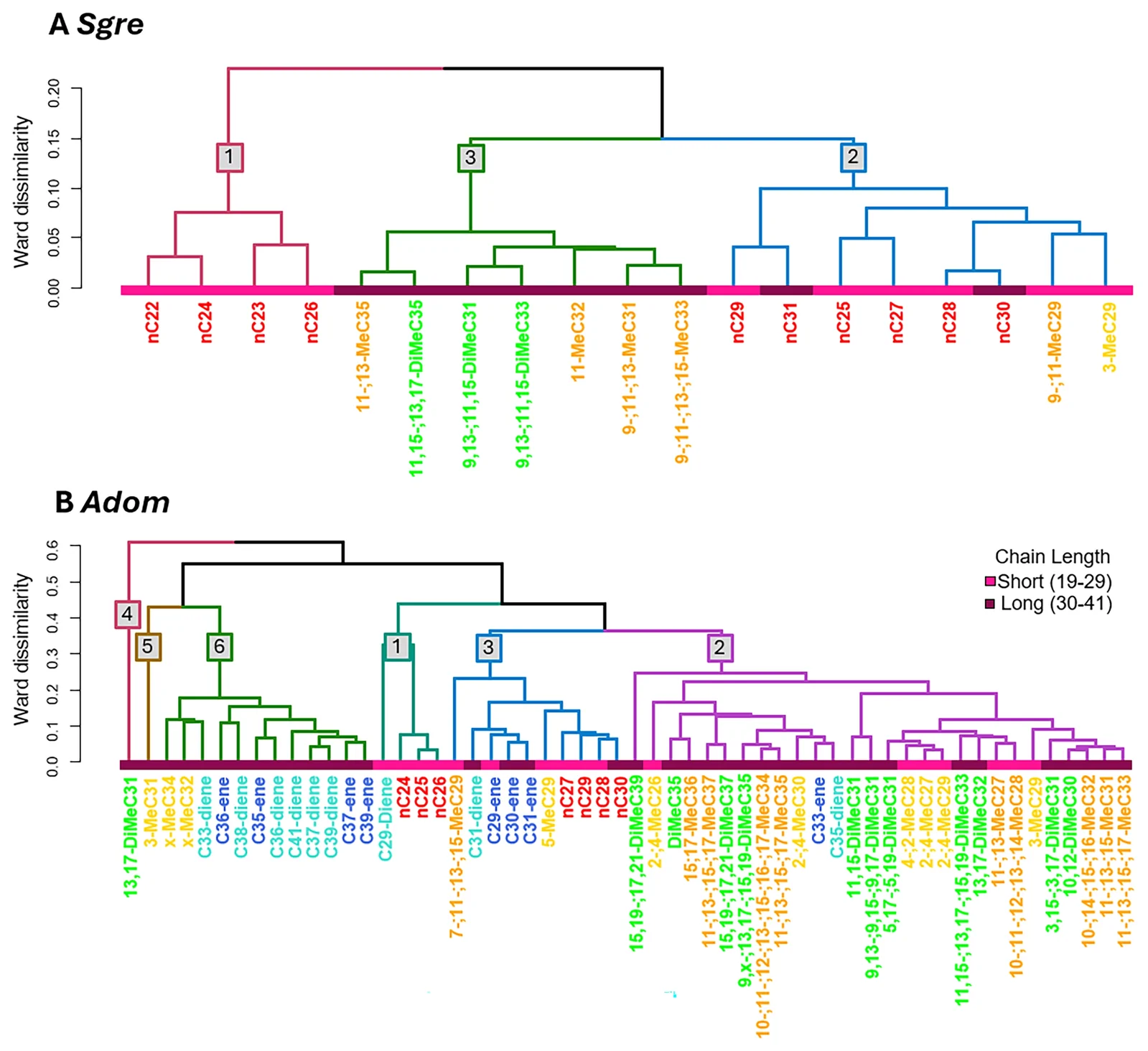
Covariation of different hydrocarbons. Hierarchical cluster analyses of the CHCs in (A) *Sgre* and (B) *Adom*, based on all samples per species. The dendrograms are based on Euclidean distances and Ward.D2 linkage of CHC profiles, cut into (A) *k* = 3 clusters for *Sgre* and (B) *k* = 6 clusters for *Adom*. Leaves represent individual CHC compounds, coloured by chemical class (*n*-alkanes: red; alkenes: royal blue; intMonoMe: orange; terMonoMe: gold; alkadienes: turquoise and DiMe: green). Branches are coloured and numbered according to the cluster assignment. Below the dendrogram, vertical bars at each leaf indicate chain-length category (short, C19–C29 and long, C30–C41).

## 4. Discussion

This study used differential extraction, varying solvent polarity and extraction duration, together with thermal acclimation to probe the spatial and chemical heterogeneity of the CHC layer in five insect species. Interactions between treatment factors were almost never significant, simplifying the interpretation of main effects. Across species, CHCs of the same structural class covaried in their proportional extraction responses, and more solid CHC classes showed higher proportional extraction with non-polar solvents and short extraction times, while more fluid classes showed the reverse. Together, these patterns are consistent with a model of spatially heterogeneous CHC organization, though the evidence is indirect and alternative explanations cannot be excluded.

### 4.1. Spatial structure: evidence from solvent effects

Across all five species, CHC class proportions in the extract varied systematically with solvent type. More solid CHC classes (*n*-alkanes and, in some species, terMonoMe) were proportionally higher in non-polar hexane extracts, while more fluid classes (intMonoMe, DiMe, alkenes and alkadienes) were proportionally higher in the more polar solvents DCM and DCMM. These patterns are broadly consistent with the ‘like dissolves like’ principle [30]: non-polar hexane shares structural similarity with straight-chain, tightly packed hydrocarbons, while the smaller and more polar DCM molecule may penetrate denser CHC aggregates more readily and interact more readily with branched or unsaturated compounds whose looser molecular packing reduces interaction specificity [8,16]. The further polarity increase in DCMM amplifies this trend for the most fluid classes in species where a monotonic gradient across solvents was observed (e.g. intMonoMe and DiMe in *S. gregaria* and *L. fuliginosus*).

However, solvent polarity alone does not fully account for all patterns. Several species showed non-monotonic or reversed responses for particular CHC classes, and the most pronounced solvent effects were observed in *S. gregaria*, which has the simplest CHC profile (lacking alkenes and alkadienes). This suggests that interspecific differences in solvent response do not only reflect the intrinsic chemical properties of the individual compounds (their polarity, degree of branching and chain length) but also species-specific intermolecular interactions and layer architecture arising from the particular combination of CHCs present. Differential solubility, matrix effects and differences in compound mobility within the layer may each contribute to the observed extraction patterns, independently of spatial organization. Solvent-dependent extraction differences are therefore consistent with CHCs of distinct phase behaviour occupying different domains, but cannot be taken as direct evidence for spatial topology.

### 4.2. Spatial structure: evidence from extraction duration

Extraction duration significantly influenced the proportional composition of CHC extracts in *L. fuliginosus*, where more solid classes (*n*-alkanes and terMonoMe) were proportionally higher in 10-minute extracts, while more fluid classes (intMonoMe, DiMe, alkenes and alkadienes) were proportionally higher in 10–10 extracts. In *F. rufibarbis*, duration effects were weak and largely non-significant. It is important to emphasize that extraction duration does not alter the CHC composition of the insect itself, but only the proportional composition of the extract changes depending on when and how long extraction takes place.

The duration-dependent separation of more solid and more fluid classes in *L. fuliginosus* is consistent with differences in extractability between CHC fractions of distinct phase behaviour: more solid, cohesive compounds may be released rapidly into solvent upon immersion, while more fluid compounds (potentially embedded within or beneath cohesive solid domains) require longer or repeated contact for efficient dissolution. If this reflects spatial organization, it would be consistent with the microscale solid–liquid heterogeneity reported by Kaneko *et al.* [25] using ATR-FTIR microspectroscopy, which found the CHC layer thickness to vary several 10s-fold across the surface, within a range of roughly 10 nm to 1 µm. However, several alternative explanations must be considered: (i) more solid CHCs may have higher intrinsic solubility in the solvents used and dissolve faster regardless of their spatial position, (ii) more cohesive domains may resist dissolution kinetically without being spatially elevated or clustered, and (iii) sequential extraction may expose compounds of lower solubility after the most soluble fraction is removed, without any spatial implication. That said, these mechanisms still imply some spatial structure: a solvent cannot selectively extract one compound class from a homogeneous, fully mixed layer except at its outermost surface, so the class-specific differences we observe point to at least partial segregation of solid and fluid compounds into distinct phases, even if that geometry cannot be resolved here. Without direct structural validation, for example via CHC topography imaging, extraction duration differences remain indirect, though convergent, evidence for spatial heterogeneity.

The weak duration effects in *F. rufibarbis* relative to *L. fuliginosus* may reflect genuine species differences in CHC layer organization, or may result from reduced statistical power in the partial design used for *F. rufibarbis* (hexane with three durations; three solvents at 10 minutes only). The absence of significant duration effects in this species should therefore be interpreted cautiously.

### 4.3. Acclimatory effects

CHC extract composition responded to temperature acclimation in species- and compound-specific ways that broadly reflect CHC biophysical properties. Under constant warm conditions (30°C), the relative proportions of more solid CHC classes, particularly *n*-alkanes, increased, which is consistent with physiological adjustment towards greater waterproofing capacity through higher saturation and chain length at elevated temperatures [3,6,18]. Conversely, more fluid classes, such as alkenes and alkadienes, showed lower relative proportions in warm-acclimatized individuals in most species, though the direction and magnitude of shifts varied considerably across species and compound classes.

Acclimation responses appeared to be less driven by CHC class identity than by the relative phase behaviour of a class within a given species’ profile, e.g. by whether a compound is relatively solid or relatively fluid compared with the other CHCs of that individual. For example, intMonoMe showed lower relative proportions at 30°C in *S. gregaria*, where they are among the more fluid compounds, but higher relative proportions at 30°C in *P. americana*, where they are relatively solid compared with the dominant alkadienes. This dependence on relative rather than absolute phase behaviour aligns with the interpretation that insects adjust the CHC layer to maintain a functionally appropriate balance of solid and fluid domains across their temperature range.

Under fluctuating temperature regimes, CHC classes with intermediate phase behaviour frequently showed the highest relative proportions, suggesting adjustment towards a layer composition that remains functional under both warm and cool conditions without becoming dysfunctionally solid or fluid [6,27]. In *S. gregaria*, where *n*-alkanes dominate, terMonoMe (an intermediate class) reached its highest relative proportions under fluctuating conditions. Similarly, in *A. domesticus*, intMonoMe, which occupy an intermediate position in that species’ phase spectrum, showed the highest relative proportions under fluctuating conditions. In *P. americana*, intMonoMe and DiMe followed a similar pattern, though interpretation is complicated by the small number of compounds present at given proportions.

An unexpected pattern was the co-occurrence of higher proportions of both *n*-alkanes and alkenes under warm conditions in *A. domesticus*, and the opposing responses to acclimation of alkenes and alkadienes between *P. americana* and *A. domesticus*. Higher relative proportions of alkenes under warm conditions have been reported in *Myrmica* and *Lasius* ants [21,34] and remain difficult to explain from a biophysical standpoint given that alkenes are typically considered more fluid compounds. One speculative possibility is that elevated alkene proportions help preserve layer fluidity at high temperature by disrupting *n*-alkane crystallization, keeping the layer from becoming overly rigid even as saturated components increase, though this remains untested. These divergent responses highlight the importance of interpreting acclimation patterns in the context of species-specific CHC profiles and ecological demands.

### 4.4. Covariation of compounds

Hierarchical cluster analyses revealed that CHC compounds grouped non-randomly by structural class and, in several species, by chain length. These patterns were consistent across acclimation treatments, indicating that covariation reflects intrinsic compound properties rather than treatment-specific artefacts. CHCs that consistently covaried across extraction conditions and acclimation regimes probably share similar biophysical properties (particularly melting point, molecular packing, cohesion and solubility) that cause them to respond similarly to changes in solvent polarity, extraction duration and temperature. Divergent patterns between compound groups indicate differences in these properties, consistent with their residing in domains of distinct phase behaviour, though this spatial interpretation remains speculative.

Within the more solid portion of the CHC layer, *n*-alkanes formed distinct clusters across most species, with shorter and longer chain-length subgroups separating in most cases except *L. fuliginosus*. Chain length determines melting point within the *n*-alkane class [15,16], and the species-dependent boundary between short and long subgroups probably marks the point at which a given chain length transitions from relatively solid to relatively fluid within that particular species’ CHC mixture. Within the more fluid portion, intMonoMe and DiMe consistently covaried across all five species, indicating shared biophysical properties, such as low melting point, owing to packing disruption from internal branching or multiple methyl groups, and probably a common domain within the CHC layer. TerMonoMe occupied an intermediate position, clustering with intMonoMe/DiMe in some species and with *n*-alkanes in others, consistent with their intermediate melting points [7].

An intriguing exception was the behaviour of alkenes and alkadienes in *A. domesticus*, where these unsaturated compounds covaried independently of the methyl-branched alkanes, suggesting a distinct domain separate from the intMonoMe/DiMe cluster. Whether this reflects genuine spatial separation, for instance an alkene-enriched domain with distinct functional properties, or simply different solubility properties cannot be determined from extraction data alone.

Covariation patterns were weakest in *P. americana*, where the CHC profile is dominated by a small number of compounds (notably a highly abundant C27-alkadiene) with most others at low relative proportions. This may limit statistical power to detect covariation or may reflect a more homogeneous CHC layer in which minor compounds are dissolved within the dominant phase rather than forming distinct domains.

In *S. gregaria*, by contrast, the absence of unsaturated compounds may produce a simpler phase landscape with clearer domain boundaries, consistent with the strongest covariation and solvent-effect patterns observed in this species.

## 5. Conclusion

This study provides indirect evidence that CHC layers are spatially and chemically heterogeneous, with CHCs of distinct phase behaviour showing differential extractability across solvents and extraction durations, consistent with an organization into domains of different physical state. This interpretation is supported by convergent patterns from multiple lines of evidence (solvent polarity effects, extraction duration effects, acclimation responses and compound covariation) but cannot be confirmed without direct structural imaging at the relevant spatial scales. Alternative explanations, including differential solubility or compound-specific diffusion kinetics, remain consistent with the data.

A key insight is that the phase behaviour of a given compound within the layer depends not on its intrinsic melting point alone, but on whether that melting point is high or low in relation to the other compounds present. For example, intMonoMe act as relatively fluid compounds in the *n*-alkane-dominated profiles of *S. gregaria* but as relatively solid compounds in the alkadiene-dominated profiles of *P. americana*, merely because of the composition of the remaining CHCs. The same principle extends beyond the cuticle: the relative fluidity of a CHC predicts which compounds transfer into the silk capture threads of spiders on contact with an insect [35]. This contextual dependence means that predictions about CHC phase organization must account for the full chemical composition of the individual's profile rather than relying on class-level generalizations. Covariation of structurally similar CHCs into shared domains, combined with the role of relative rather than absolute phase behaviour, suggests that the CHC layer possesses functional modularity: groups of compounds with similar biophysical properties aggregate together, and their proportional composition can be adjusted through biosynthetic plasticity in response to temperature, while the functional architecture (solid domains providing waterproofing, fluid regions enabling semiochemical diffusion) is maintained across conditions. In this way, the complex composition of CHC layers may allow simultaneous optimization of multiple functions while avoiding the need for trade-offs [36]. Our results support the candidate organization introduced in figure 1 and allow us to refine it into a single model (figure 6): the more-solid compounds (*n*-alkanes and terMonoMe) may co-aggregate into discrete solid domains embedded within a more-fluid matrix—itself differentiated into a high-viscosity and a low-viscosity fraction—that overlies the cuticle. Our extraction data provide evidence on the chemical composition and co-aggregation of these phases but not on their precise vertical arrangement: the solid domains may lie level with the liquid surface, protrude above it or be fully embedded as detached inclusions (figure 6A–C). Our extraction approach sampled CHCs from the entire body surface, so the spatial organization inferred here represents a whole-body average. While most body parts are chemically quite similar [37], whether our suggested spatial organization holds across individual body parts, cuticle regions of differing hardness or texture or functionally specialized areas remains unknown and warrants targeted investigation. Future work should also address the functional consequences of stronger versus weaker phase separation, the adaptive value of CHC heterogeneity across ecological gradients and whether the spatial organization inferred from extraction behaviour can be confirmed and spatially resolved through direct imaging approaches.

**Figure 6.**
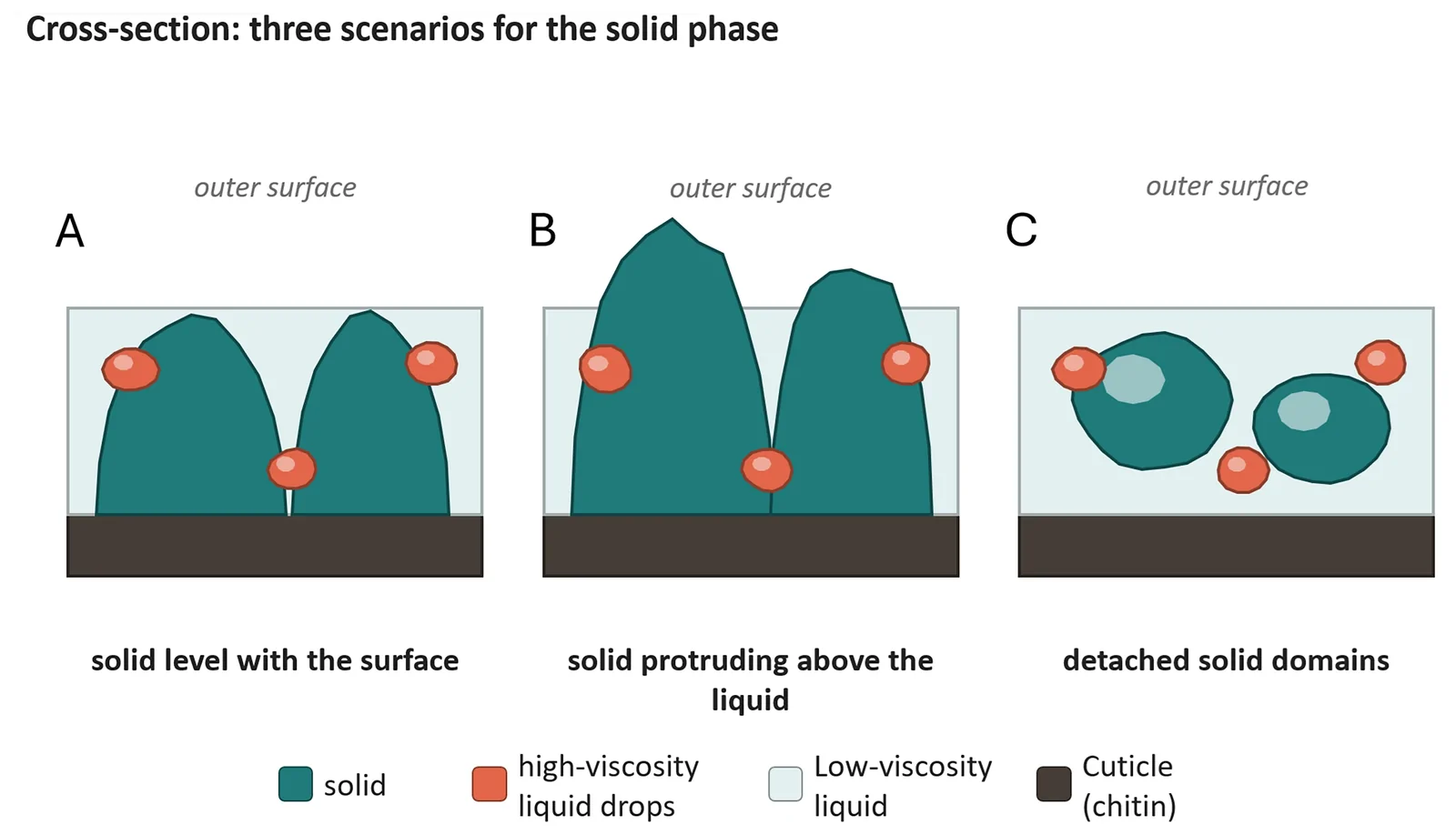
Proposed organization of the CHC layer (three candidate geometries). Schematic cross-section (cuticle at the bottom and outer surface at the top): more-solid compounds (*n*-alkanes, terminally branched alkanes; teal) form discrete solid domains within a more-fluid matrix comprising a viscous fraction (orange droplets) and a thin-liquid fraction (pale) over the cuticle (dark brown). Panels show the three vertical arrangements our data cannot distinguish: (A) solid level with the surface, (B) solid protruding above the liquid and (C) detached domains. Schematic; not to scale.

## Supplementary Material



## Data Availability

Data and code can be accessed via Zenodo [38]. Supplementary material is available online [39].
