# Supplementary material for: Spatial and chemical heterogeneity of insect cuticular hydrocarbon layers: insights from extraction methods and acclimation regimes
Source: R Soc Open Sci. 2026 Nov 4;13(11):261519. doi: 10.1098/rsos.261519 (PMC13730641; doi:10.1098/rsos.261519)
Supplement: Supplementary file 1 [file rsos.261519.zip › rsos.261519/rsos261519_si_001.pdf]

# Electronic supplementary material

## Spatial and chemical heterogeneity of insect cuticular hydrocarbon layers: insights from extraction methods and acclimation regimes

Sascha Schlüter*, Kiara Mangin, Florian Menzel

Institute of Organismic and Molecular Evolution, Faculty of Biology, Johannes-Gutenberg-Universität Mainz, Germany

Royal Society Open Science

DOI: 10.1098/rsos.261519

**Figure S1: Covariation of different hydrocarbons.** Hierarchical cluster analyses of the CHCs in (A) *Pame*, (B) *Lful*, and (C) *Fruf*, based on all samples per species. The dendrograms are based on Euclidean distances and Ward.D2 linkage of CHC profiles, cut into k = 3 clusters for *Pame* (A) and k = 6 clusters for *Lful* (B) and *Fruf* (C). Leaves represent individual CHC compounds, coloured by chemical class (*n*-alkanes: red; alkenes: royalblue; internally branched monomethylalkanes [intMonoMe]: orange; terminally branched monomethylalkanes [terMonoMe]: gold; alkadienes: turquoise; dimethylalkanes [DiMe]: green). Branches are coloured and numbered according to the cluster assignment. Below the dendrogram, vertical bars at each leaf indicate chain-length category (short, C19–C29; long, C30–C41.

Fig. S1

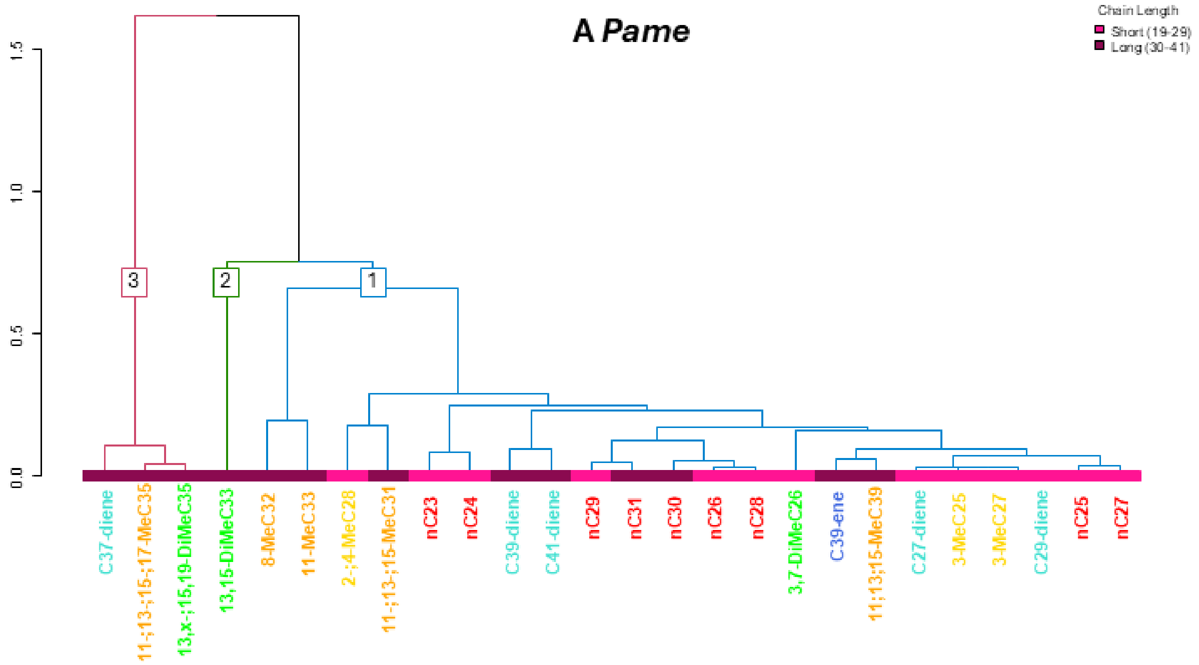

A Pame
Chain Length
Short (19-29)
Long (30-41)
1.5
1.0
0.5
0.0
3
2
1
C37-diene
11-;13-;15-;17-MeC35
13,x-;15,19-DiMeC35
13,15-DiMeC33
8-MeC32
11-MeC33
2-;4-MeC28
11-;13-;15-MeC31
nC23
nC24
C39-diene
C41-diene
nC29
nC31
nC30
nC26
nC28
3,7-DiMeC26
C39-ene
11;13;15-MeC39
C27-diene
3-MeC25
3-MeC27
C29-diene
nC25
nC27


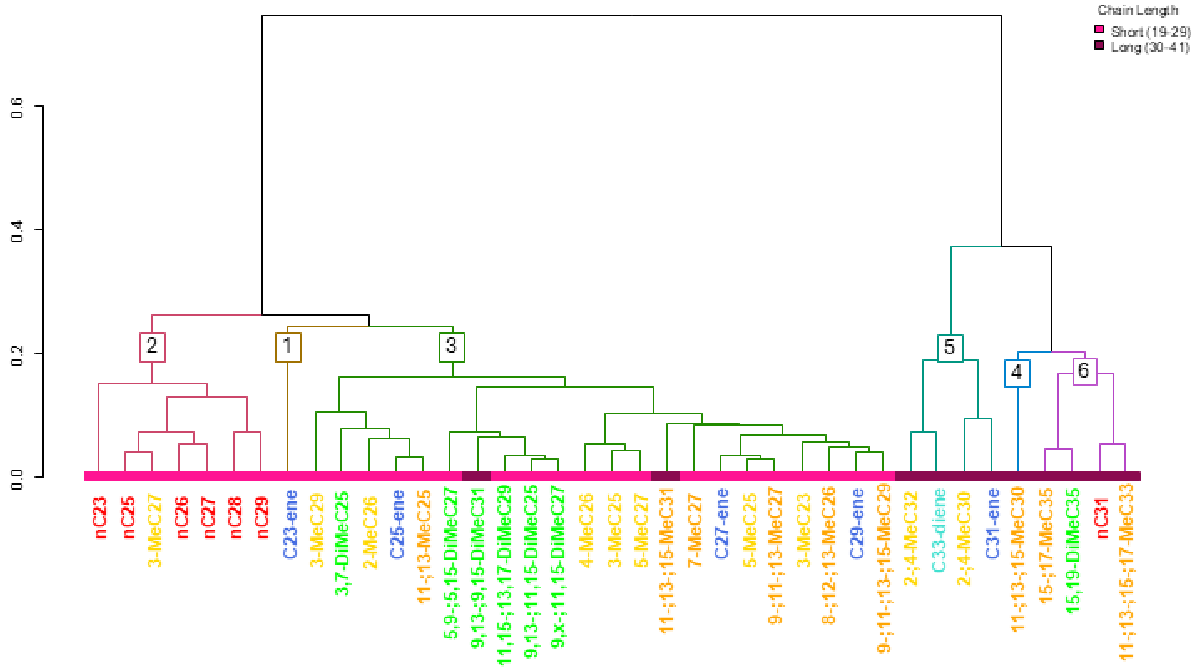

B Lful
Chain Length
Short (19-29)
Long (30-41)
0.6
0.4
0.2
0.0
2
1
3
5
4
6
nC23
nC25
3-MeC27
nC26
nC27
nC28
nC29
C23-ene
3-MeC29
3,7-DiMeC25
2-MeC26
C25-ene
11-;13-MeC25
5,9-;5,15-DiMeC27
9,13-;9,15-DiMeC31
11,15-;13,17-DiMeC29
9,13-;11,15-DiMeC25
9,x-;11,15-DiMeC27
4-MeC26
3-MeC25
5-MeC27
11-;13-;15-MeC31
7-MeC27
C27-ene
5-MeC25
9-;11-;13-MeC27
3-MeC23
8-;12-;13-MeC26
C29-ene
9-;11-;13-;15-MeC29
2-;4-MeC32
C33-diene
2-;4-MeC30
C31-ene
11-;13-;15-MeC30
15-;17-MeC35
15,19-DiMeC35
nC31
11-;13-;15-;17-MeC33

Fig. S1 (continued)

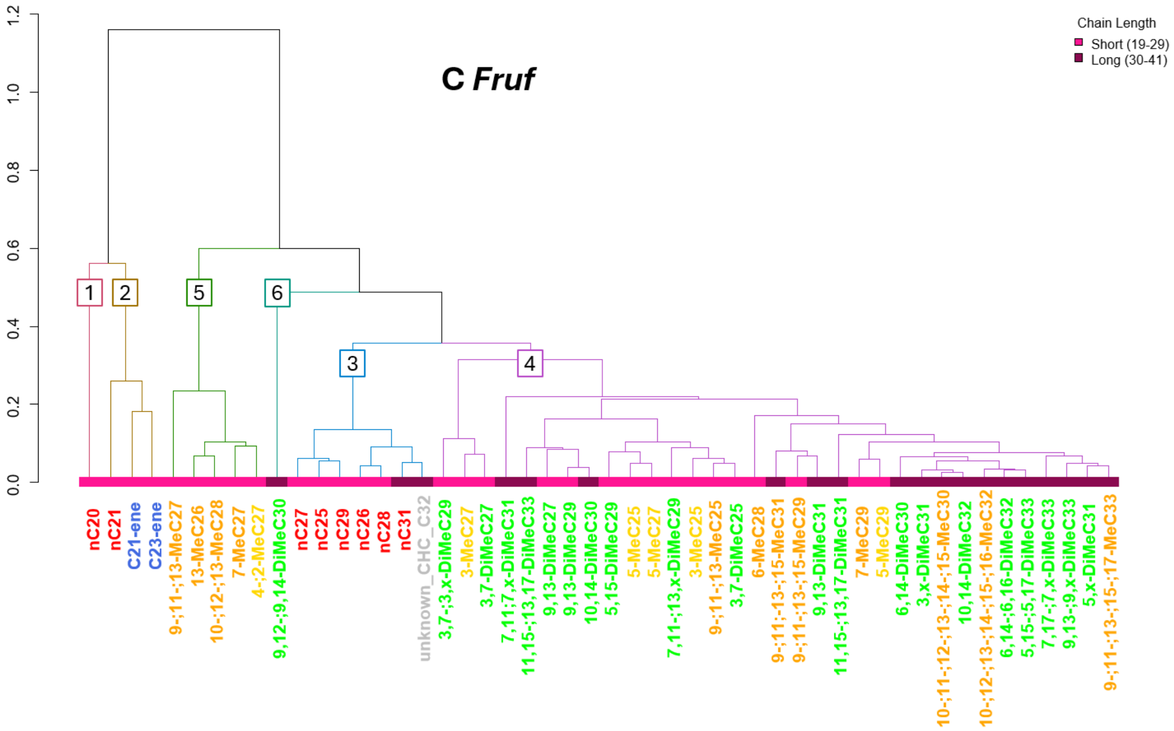

**Figure S2. Cuticular hydrocarbon (CHC) class composition separates the five species.** Non-metric multidimensional scaling (NMDS) ordination of all individuals (N = 338), based on Bray–Curtis dissimilarities computed from the proportional abundances of the six CHC classes only (*n*-alkanes, terminally and internally methyl-branched alkanes, dimethylalkanes, alkenes, and alkadienes); individual compounds and chain length were collapsed into these class proportions, so the ordination reflects class-level composition rather than the full CHC profile. Each point is one individual, colour-coded by species: *Sgre*, *Schistocerca gregaria*; *Adom*, *Acheta domesticus*; *Pame*, *Periplaneta americana*; *Lful*, *Lasius fuliginosus*; *Fruf*, *Formica rufibarbis*. Individuals closer together have more similar CHC-class compositions. Individuals formed tight species clusters, and species identity explained 87% of the variation in composition (PERMANOVA on Bray–Curtis distances, pseudo-$F_{4,333}$ = 542, $R^2$ = 0.87, p = 0.001). The partial overlap of *A. domesticus* and *L. fuliginosus* reflects similar class-level proportions and does not imply similar CHC profiles, since the underlying compounds and chain lengths differ markedly and are not represented here. Two-dimensional stress = 0.12.

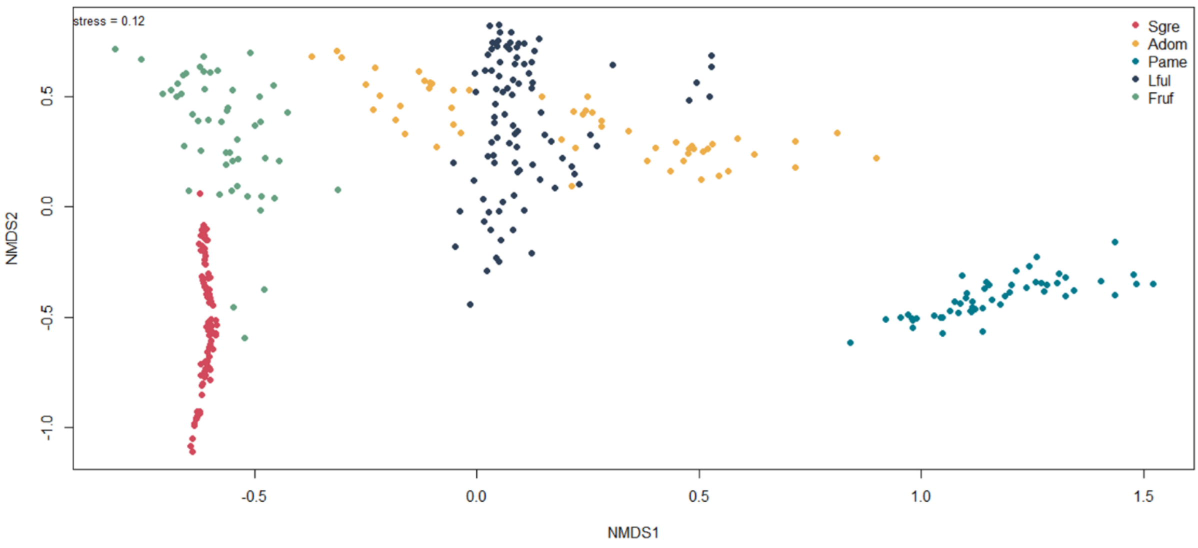

**Table S1: Inter-individual variability of cuticular hydrocarbon (CHC) class proportions within treatment groups.** For each species, the relative abundance of each CHC class (proportion of total CHC peak area per individual) was quantified, and its variation among individuals was assessed separately within each treatment group (acclimation × solvent for *Schistocerca gregaria*, *Acheta domesticus* and *Periplaneta americana*; solvent × extraction time for *Lasius fuliginosus* and *Formica rufibarbis*). Each tabulated value is the coefficient of variation (CV = 100 × SD/mean, %) of a class's proportion among the individuals of a treatment group, averaged over the treatment groups in which that class reached at least 0.5% mean abundance (all treatment groups comprised ≥ 3 individuals) Computing the CV within treatment groups isolates inter-individual variability from the experimental treatments, so the values reflect how much individuals of the same species differ from one another. n, number of individuals analyzed per species. "NA" denotes a class that was absent or remained below the 0.5% threshold in every treatment group of that species. Pooled across all species and classes, the median CV was 31% (interquartile range 21–38%), indicating low-to-moderate within-group variability; the few high values (e.g. alkadienes in *L. fuliginosus*, alkenes in *F. rufibarbis*) correspond to classes present only at trace abundance, where the small mean inflates the CV. Class abbreviations: terMonoMe, terminally methyl-branched alkanes; intMonoMe, internally methyl-branched alkanes; DiMe, dimethylalkanes.

| species | n | n-alkanes | terMonoMe | intMonoMe | DiMe | alkenes | alkadienes |
|---|---|---|---|---|---|---|---|
| ***Sgre*** | 90 | 13 | 22 | 32 | 29 | NA | NA |
| ***Adom*** | 54 | 35 | 15 | 30 | 34 | 39 | 47 |
| ***Pame*** | 54 | 21 | 11 | 50 | NA | 33 | 5 |
| ***Lful*** | 90 | 45 | 21 | 22 | 34 | 20 | 170 |
| ***Fruf*** | 50 | 70 | 32 | 25 | 26 | 231 | NA |
